# The Effect of Tomato Pomace on the Oxidative and Microbiological Stability of Raw Fermented Sausages With Reduced Addition of Nitrites

**DOI:** 10.1155/ijfo/6146090

**Published:** 2025-03-25

**Authors:** Patrycja Skwarek, Małgorzata Karwowska

**Affiliations:** Department of Animal Food Technology, Sub-Department of Meat Technology and Food Quality, University of Life Sciences in Lublin, Lublin, Poland

**Keywords:** carbonyl groups, oxidative stability, raw fermented sausages, tomato pomace

## Abstract

The aim of the study was to assess the impact of the addition of freeze-dried tomato pomace (TP) on the physicochemical parameters, oxidative changes, and microbiological stability of raw fermented sausages with reduced addition of nitrites. It was shown that the addition of TP reduced the pH value of experimental meat products. All analyzed meat products were characterized by similar low water activity in the range of 0.842–0.865. The addition of TP also increased the antioxidant activity of sausages, which effectively inhibited oxidative changes in lipids of meat products by reducing the TBARS values. The use of 1.5% and 2.5% TP also resulted in an increase of redness (*a*^∗^) of the sausages, which may have a positive impact on its acceptance by consumers. Additionally, sausages containing TP were characterized by a higher heme iron content as well as higher carbonyl groups. The addition of TP did not affect the number of lactic acid bacteria in sausages. The most promising results were obtained for dry fermented sausage with 2.5% addition of TP. It can therefore be concluded that the use of TP as a natural antioxidant makes it possible to reduce nitrates in the production of meat products and additionally helps to reduce food waste.

## 1. Introduction

Meat consumption has long been an important component of the human diet and is considered necessary for the proper development of the human body [1]. It is an important source of energy and a number of nutrients, including high-quality protein, minerals, and vitamins [2]. Nowadays, many types of meat products are produced. One of them are raw fermented sausages; their consumption is constantly increasing, mainly due to their unique sensory properties. Moreover, fermented sausages are considered a healthy and safe product that has a beneficial effect on humans [3]. This is mainly related to their unique nutritional composition and health-promoting properties resulting from the fermentation process. They are an excellent source of high-quality protein that contains all the essential exogenous amino acids. Additionally, they contain saturated and unsaturated fatty acids, mainly oleic acid and polyunsaturated fatty acids omega-3 and omega-6, which have a beneficial effect on the human body, lowering LDL cholesterol levels, thus supporting the circulatory system. In addition, fermented meat products are rich in essential microelements, including B vitamins, as well as minerals (heme iron, zinc, selenium, phosphorus), crucial in protection against oxidative stress [4]. Moreover, bacteria, mainly *Lactobacillus*, *Pediococcus*, and *Staphylococcus*, also play an important role during the fermentation process, which can support the intestinal microflora and improve digestion. Due to the fact that they lower the pH of raw fermented products and participate in the transformation of proteins and fats during proteolysis and lipolysis processes, they may affect the safety, taste, texture, and shelf life of the finished product. In the production of raw fermented sausages, starter cultures are often used to ensure a controlled fermentation process, which is crucial for stabilizing the characteristic red color, limiting the development of undesirable bacteria and molds, and the development of flavor compounds [5]. However, due to the relatively high level of fat and the characteristic processing technology, fermented sausages are susceptible to lipid and protein oxidation and the growth of undesirable bacteria [6]. These reactions are one of the main causes of deterioration of the quality of meat and meat products because they affect their color, taste, consistency, and nutritional value and thus also contribute to shortening their shelf life [7]. Lipid oxidation is also responsible for the formation of off-flavors in meat products (including rancid taste) and also affects the texture and digestibility of proteins [8, 9]. Lipid oxidation occurs as a result of a reaction of free-chains that generate many by-products, particularly aldehydes. Malondialdehyde (MDA), one of the most closely studied aldehydes, is considered an indicator of oxidative damage in fats [10]. Protein is also an important ingredient in meat. Moderate protein degradation during the processing and storage of meat products can help improve their taste and nutritional value. However, excessive oxidation of proteins can lead to deterioration of the quality of the meat product, negatively affecting its texture, color, and taste. Protein oxidation is a free radical chain reaction, similar to lipid oxidation. One of the most commonly observed effects of protein oxidation in meat is the formation of carbonyl compounds [11].

However, the rate of oxidation processes can be effectively slowed down by using antioxidants. Nitrogen compounds are one of the most popular additives used in the meat industry which have, among others, antioxidant properties. In the curing process, they are mainly used to improve the shelf life of the product, as they effectively inhibit the growth of many harmful microorganisms [12]. Thanks to their antimicrobial properties they effectively inhibit the growth of *Clostridium botulinum* and *Staphylococcus aureus* (STA) bacteria. Through their action, they inhibit their metabolism and the production of botulinum toxin and toxins responsible for food poisoning, protecting the product and ensuring its microbiological safety [13]. In addition to their preservative effect, their use also contributes to the development of the characteristic red–pink color and taste of processed meat [14]. Moreover, stable complexes between nitrite-derived compounds and heme iron inhibit the release of free iron, which is therefore not available to initiate lipid peroxidation [15]. According to Safa et al. [16], nitrogen compounds significantly delay the formation of carbonyl compounds responsible for the rancid taste. However, nitrates and nitrites are among the most frequently questioned food additives by consumers [17]. High levels of these substances in the human body can lead to methemoglobinemia, a condition in which hemoglobin is converted to methemoglobin, thereby losing its ability to transport oxygen, which can be fatal in severe cases [18]. Nitrite, particularly in an acidic environment, often reacts with various components of meat, such as amino acids, myoglobin, and phenolic compounds. Additionally, as a nitrosation agent, it reacts with secondary amines to form highly carcinogenic nitrosamines [19]. Furthermore, nitrite can reduce the absorption of proteins and fats from food, which may lead to thyroid dysfunction [20]. It is therefore important to remember that the European Union has established rules on maximum levels of nitrites in meat products to protect the health of consumers while ensuring their effectiveness in preventing the development of pathogens. According to Commission Regulation (EC) No. 1129/2011 on food additives, sodium nitrite (E250) in raw fermented meat products cannot be used in an amount greater than 150 mg/kg of the finished product (Commission Regulation (EU) No. 1129/2011 [21]). Therefore, a challenge for the meat industry is to develop methods to reduce these additives in meat products to limit their consumption [22]. Nowadays, the strategies used are mainly based on improving the composition of meat products by including bioactive ingredients, reducing the amount of exogenous additives and native harmful compounds.

One of the more and more commonly used methods is the use of natural plant raw materials, which allow the reduction/elimination of nitrates from the final product, while achieving the same or similar effects in meat products. Moreover, their use also has a beneficial effect on increasing the antioxidant properties of meat products due to their strong antioxidant properties [23]. An example of this is the use of by-products such as fruit and vegetable pomace, including tomato pomace (TP). Promising results from various studies suggest that using tomato by-products as natural additives can help extend the shelf life of meat products, offering consumers food with only natural ingredients. The use of these additives often leads to improvements in nutritional value, reduced lipid oxidation, and increased stability of the shelf life, while maintaining or even enhancing sensory properties and overall acceptability [24]. Tomatoes characterized excellent antioxidant properties and intense red color, including the presence of lycopene; they can be utilized in the meat industry to prevent oxidative degradation and discoloration, as well as to produce functional foods.

Research conducted as part of the previous experiment [25] confirmed the benefits of using TP as an addition to raw ripening products. The results obtained inspired further research on the use of higher levels of TP and their impact on the oxidative stability of sausages. In this context, the aim of study was to analyze the effect of freeze-dried TP in the amount of 1.5% and 2.5% on the physicochemical parameters, amino acid profile, antioxidant properties, and oxidative changes occurring in raw-ripened sausages with reduced nitrate content, both after production and after 3 months of storage. Additionally, microbiological analyses were also carried out to determine product safety and storage stability.

## 2. Material and Methods

### 2.1. Dry Fermented Sausage Production

**Figure 1:**
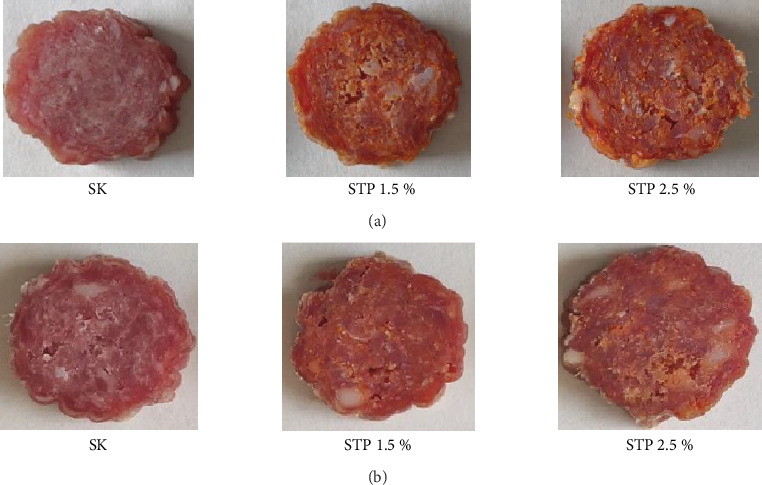
Cross-sectional appearance of fermented sausages (a) after the production and (b) after 3 months of storage (90 days). SK—control sample; STP 1.5%—sample with 1.5% addition of TP; STP 2.5%—sample with 2.5% addition of TP.

The study analyzed dry fermented sausages produced with an addition of 50-mg sodium nitrite per kilogram of meat. The sodium nitrite concentration was reduced compared to the maximum permissible levels (150-mg sodium nitrite per kilogram of meat) specified in Commission Regulation (EU) No. 1129/2011 [21]. The sausages were made using ham and back fat from Polish large white pigs, obtained from a local slaughterhouse 48 h postmortem. The raw materials were transported to the laboratory under refrigeration conditions. The research was conducted at the Department of Meat Technology and Food Quality at the University of Life Sciences in Lublin under semitechnical conditions. The meat and fat were ground using a commercial grinder (model KU2-3EK, Mesko-AGD Skarżysko-Kamienna, Poland) with a grinding plate of 0.01-m diameter. The dry fermented sausages were made using a mixture of 85% meat and 15% fat. Each batch of the sausage mixture was added with 0.6% glucose and 2.8% curing mixture, which consisted of sea salt and sodium nitrite. This formulation ensured a sodium nitrite concentration in the mixture of 50 mg/kg. Commercial starter cultures (Moguntia, Bessa START) were also added. According to the manufacturer's declaration, the mixture included *Staphylococcus xylosus* and *Pediococcus pentosaceus*. They were used in an amount of 30 g per 50 kg of meat raw material. The study also used lyophilized TP (seeds and skins), which was lyophilized at −50°C in a lyophilizer (Labconco Free-Zone, United States) and ground into particles smaller than 0.3 mm just before use. Three sausage variants were prepared: SK—control sample, STP 1.5%—sample with 1.5% TP addition, and STP 2.5%—sample with 2.5% TP addition. After mixing the ingredients in a universal machine model KU2-3EK (Mesko-AGD, Skarżysko-Kamienna, Poland) with a R4 type mixer (100 rpm, 3 min), the raw mixture was stuffed into fibrous casings (ø 65 mm; Viskase, Chicago, United States) using a manual sausage stuffer. The sausages weighed approximately 500 g. In the subsequent stages, the sausages were placed in fermentation chambers (model ITALFROST-DE RIGO-GS, Pszczyna, Poland), where the fermentation process continued until a weight loss of 30 ± 3% was achieved, which took 20 days. The fermentation process was divided into three phases: Stage 1—temperature 20°C–22°C, relative humidity 55%–65%, for 3 days; Stage 2—temperature 14°C–16°C, relative humidity 68%–75%, for 3 days; Stage 3—temperature 13°C, relative humidity 76%, for 14 days. The groups of dry fermented sausages are depicted in Figure 1. The measurements were conducted after production (Day 0) and again following a storage period of 3 months (90 days).

### 2.2. Proximate Chemical Composition

The moisture, protein, collagen, and fat content of dry fermented sausages was measured on samples weighing approximately 200 g after grinding, using a Food Scan Lab 78,810 analyzer (Foss Tecator Co., Ltd., Hillerod, Denmark).

### 2.3. The Physicochemical Parameters (pH and Water Activity)

The pH value was determined in homogenates obtained by homogenizing 10 g of the product sample with 50 mL of distilled water for 1 min. The pH measurement was performed using a digital pH meter CPC-501 (Elmetron, Zabrze, Poland), equipped with a temperature sensor and a pH electrode (model ERH-111, Hydromet, Gliwice, Poland). Before starting the analysis, the pH meter was calibrated with buffer solutions of pH 4.0, 7.0, and 9.0. Water activity (aw) was measured using a water activity analyzer (Novasina AG, Lachen, Switzerland), which was calibrated with Novasina SAL-T humidity standards corresponding to relative humidities of 33%, 75%, 84%, and 90%. A 5-g sample was placed in the device's chamber, and the measurement was carried out automatically at a temperature of 20°C.

### 2.4. Antioxidant Activity

The measurement was conducted on experimental sausages according to the method previously described by Skwarek and Karwowska [25]. The samples' ability to scavenge ABTS⁣^∗^+ and DPPH free radicals was assessed by comparing the results to a Trolox standard curve. The results were expressed in milligrams per gram of product.

### 2.5. Oxidation Stability (Thiobarbituric Acid Reactive Substances (TBARS) Values)

The lipid oxidation process was analyzed using the method for measuring substances reactive to 2-thiobarbituric acid (TBARS). The quantification of compounds reacting with thiobarbituric acid (TBA) was conducted following the procedure described by Pikul et al. [26], with perchloric acid used as the solvent. Absorbance was measured at 532 nm using a UV spectrophotometer (Nicolet Evolution 300, Thermo Electron Corp., Waltham, Massachusetts, United States). The results were presented as milligram of MDA per kilogram of sample.

### 2.6. Protein Carbonyl Content

Protein carbonyl content was measured using the MAK094 kit (Sigma-Aldrich, St. Louis, Missouri, United States) designed for detecting carbonyls in proteins, following the procedures outlined in the technical bulletin. The results are presented as nanomoles of carbonyls per milligram of protein.

### 2.7. Amino Acid Content

The content of amino acids (mg/g^−1^) was analyzed as indicators of the progressive change of proteolytic proteins in dry fermented sausages. The content of free amino acids was determined using the method described by Stadnik and Dolatowski [27]. The analysis was carried out using an automatic amino acid analyzer AAA 400 (Ingos Ltd., Czech Republic), equipped with an ion-exchange column Ostion LG ANB (36 × 0.37 cm) operating at 70°C. The following amino acids present in dry fermented sausages were analyzed: asparagine (Asn), threonine (Thr), serine (Ser), glutamine (Glu), proline (Pro), glycine (Gly), alanine (Ala), valine (Val), isoleucine (Ile), leucine (Leu), tyrosine (Tyr), phenylalanine (Phe), histidine (His), lysine (Lys), and arginine (Arg).

### 2.8. Microbiological Analyses

Microbiological tests were conducted to determine the number of lactic acid bacteria (LAB), Enterobacteriaceae (EB), *Escherichia coli* (EC), yeasts and molds (YM), and STA. An automatic microbial counting system, TEMPO LAB (Biomerieux, TEMPO System, Marcy l'Etoile, France) was used for this purpose. Microbiological determinations were performed using original TEMPO tests: TEMPO LAB for LAB, TEMPO EB for EB, TEMPO EC for EC, TEMPO YM for YM, and TEMPO STA for STA. Sample incubation conditions were as follows: for LAB, incubation time was 40–48 h at 37°C; for EB and EC, 22–27 h at 35°C; for YM, 72–76 h at 25°C; and for STA, 24–27 h at 37°C. The results were expressed as the logarithm of colony-forming unit per gram of product (log CFU/g).

### 2.9. Color Measurements

The color of dry fermented sausages was analyzed using the *L*^∗^, *a*^∗^, and *b*^∗^ parameters (*L*^∗^—lightness, *a*^∗^—redness, *b*^∗^—yellowness) with an X-Rite 8200 colorimeter (X-Rite, Inc., Grand Rapids, Michigan, United States). Color measurements were performed on samples with 5-cm thick and a depth of 20 mm [28]. Before measurements, the colorimeter was calibrated using the provided black and white plates. The measurement was made on the cross section immediately after cutting. The instrumental settings included using a 12-mm diameter aperture, a D65 light source, and a 10° standard colorimetric observer. The color difference (Δ*E*) between the control and test samples during storage was calculated according to AMSA [28] using the following formula:

 $$\begin{array}{c} \Delta Ε=\sqrt{{\Delta L}^{2}+{\Delta a}^{2}+\Delta b^{2}.} \end{array}$$

For interpreting the results, the following criteria were applied: 0 < Δ*E* < 1, the difference is not noticeable to the observer; 1 < Δ*E* < 2, the difference can only be perceived by an experienced observer; 2 < Δ*E* < 3.5, the difference is visible to both experienced and inexperienced observers; 3.5 < Δ*E* < 5, the color difference is clearly noticeable; Δ*E* > 5, the observer perceives two distinctly different colors [29].

### 2.10. Heme Iron Content

The heme iron content in the samples was determined using the method proposed by Hornsey [30]. Absorbance measurements were performed with a UV-VIS spectrophotometer model Nicolet Evolution 300, manufactured by Thermo Electron Corp., Waltham, Massachusetts, United States. The total pigment and heme iron content in the samples was calculated according to the procedure described by Lee et al., with results expressed in mg/kg^−1^.

### 2.11. Instrumental Evaluation of the Texture

The texture parameters were analyzed using the TA.XT2 Texture Analyzer (Stable Micro Systems Ltd., Godalming, United Kingdom). Cylindrical samples of meat products, with a diameter of 20 mm and a length of 20 mm, were used for the test. The test was conducted at a speed of 10 mm/min, with a compression level of 50% of the initial sample height. Measurements were carried out at a temperature of 20°C–22°C. Hardness (Newton) was determined from the force–time curve obtained during the test.

### 2.12. Statistical Analysis

The experiment was conducted twice. The collected data were analyzed using Statistica 9.1 (StatSoft, Poland). Results are presented as means ± standard deviations, with each sample analyzed in triplicate. The normality of the distribution of variables in each group was assessed using the Shapiro–Wilk test. Effects between categorical factors (storage time and type of sausage) and variables among subgroups were analyzed using factorial ANOVA. Homogeneous groups were identified using the post hoc HSD Tukey test. A significance level of *p* < 0.05 was adopted, indicating statistically significant differences.

## 3. Results

### 3.1. Proximate Chemical Composition of Fermented Sausage

The experimental fermented sausages with TP were characterized by a high protein content (41.89%–44.31%) as shown in Table 1. The statistical analysis did not reveal statistically significant differences in fat, protein, and moisture content (*p* ≤ 0.05) between the sausage variants both after production and after 90 days of storage. In the case of these ingredients, no significant effect of storage time was found. A significant difference in the collagen content was observed between the control sample analyzed after the production process and the control sample analyzed after 90 days of refrigerated storage, but no statistical differences in this parameter were observed within the variable on the same day. The salt concentration in the three groups of fermented sausages was similar due to the same amount added during production, although the samples with the addition of TP were characterized by a slightly lower concentration of this ingredient. However, this phenomenon could be caused by the large amount of sugars in the TP.

### 3.2. Results of pH and Water Activity

Table 2 shows the pH and water activity values of raw fermented sausages with different concentrations of TP. Statistical analysis showed a significant impact of the concentration of TP and storage on the pH of fermented sausages. The increase in the percentage of TP significantly reduced the pH of the sausages. At the same time, the pH of the samples with the addition of TP was significantly higher compared to the samples after production (0 day). Statistical analysis showed a significant effect of TP addition and no effect of storage on the water activity of fermented sausages. After the production, the sample with 2.5% TP had the highest water activity.

### 3.3. Antioxidant Activity and Oxidative Stability of Raw Fermented Sausages

The results of measuring the antioxidant activity of sausages showed statistically significant differences (*p* ≤ 0.05) between the samples (Table 3). The samples with the addition of 2.5% TP were characterized by the highest antioxidant activity both against the ABTS+ radical and DPPH. It was also found that the antioxidant activity of the samples was significantly higher in sausages analyzed after 90 days of storage compared to samples tested after production.

Statistical analysis showed a significant impact of TP addition and storage on the TBARS value. The effect of the addition of TP on the reduction of secondary fat oxidation products was found. It was also observed that with increasing TP concentration, less oxidative degradation of meat products occurred. After 90 days of storage, the samples were characterized by significantly lower TBARS compared to the results obtained on Day 0.

### 3.4. Amino Acid Content in Raw Fermented Sausages

Table 4 shows the amino acid profile of fermented sausages. There was no significant effect (*p* ≤ 0.05) of TP on the amino acid profile of sausages. Statistical analysis showed significant differences in the content of Ala, Pro, Arg, and Leu in the control sample on the 0 and 90 days. The control sample was characterized by significantly higher Ala and Leu content on Day 0 compared to Day 90. A similar phenomenon was observed for the sample with 1.5% TP in the case of Pro and the sample with 2.5% for Arg.

### 3.5. Results of Microbiological Analyses

Statistical analysis of the results of microbiological analyses (Table 5) did not show a significant effect of the TP addition on the number of LAB and YM. However, a significant effect of storage was found, as products with TP on Day 90 were characterized by a significantly lower LAB compared to Day 0. The number of LAB in the analyzed products ranged from 6.80 to 8.60 log CFU g^−1^. None of the sausages contained EC, EB, or STA.

### 3.6. Color Parameters, Heme Iron Content, and Hardness

The *L*^∗^, *a*^∗^, and *b*^∗^ values of sausage samples with the addition of TP are presented in Table 6. It was found that the level of TP addition and storage had a statistically significant (*p* ≤ 0.05) effect on the tested color parameters. The increase in the concentration of the TP resulted in a decrease in lightness in the analyzed meat products. Additionally, it was observed that during storage, the lightness value decreased for all variants of experimental sausages. The influence of the TP level and storage time was observed for the color parameter *a*^∗^ and *b*^∗^. Sausages without plant additives had the lowest redness. The addition of TP resulted in a significant increase of redness in the sausages compared to the control sample. In the case of the *b*^∗^ parameter, the same tendency was observed, which could be due to the presence of a large amount of carotenoids in TP. Color differences (Δ*E*) between samples of raw fermented sausages with different amounts of TP showed that the greatest color changes occurred in the case of sausages with the highest TP content. Obtained Δ*E* values greater than 5 mean that the observer notices two different colors of the samples.

Statistical analysis showed significant differences in the content of heme iron in raw-ripened sausages. The addition of TP had a significant impact on the increase in the heme iron content of the samples. The sample with 2.5% TP had the highest value of this parameter. Statistically significant differences (*p* ≤ 0.05) were also observed in terms of storage. All samples analyzed after production were characterized by a higher content of heme iron compared to sausages tested after 90 days of refrigerated storage.

The texture measurement results showed that the addition of TP had a significant effect (*p* ≤ 0.05) on the hardness of the tested sausages. It was found that as the concentration of TP increases, the hardness of meat products increases, which may be related to the gelling properties of proteins present in TP, thus causing chemical retention of water in the protein matrix and an increase in the hardness of raw fermented sausages.

## 4. Discussion

The meat industry needs effective and safe solutions to inhibit the oxidation processes of ingredients of meat products using natural ingredients to replace chemical additives. Currently, much attention is paid to the use of by-products from fruit and vegetable processing as valuable bioactive ingredients. The results of the experiment performed in this study showed a significant impact of the addition of TP (1.5%–2.5%) on selected parameters of raw fermented sausages. It was found that the addition of TP had no effect on the fat and protein content in meat products, even though TPs are rich in proteins (34%) and lipids (30%) [31]. The results obtained were similar to those published in previous work [25]. The observed differences in collagen content between the control sample analyzed after the production process compared to those analyzed after 90 days of storage could be mainly due to the enzymatic degradation of collagen and the hydrolysis processes that occur during maturation and storage. Sausages with TP, which contain high amounts of lycopene capable of neutralize free radicals, as well as responsible for the breakdown of collagen and other structural proteins, could slow the loss and degradation of collagen as a result of their antioxidant action, thereby contributing to prolonging the shelf life and improving the texture of the finished meat product [24].

Statistical analysis showed that the use of TP had an impact on the physicochemical properties of the tested sausages. The pH of meat products was below 5.0, which is an important factor inhibiting the development of pathogenic microorganisms [32]. Similar to our experiment, other researchers [33–35] confirmed that addition of TP to meat products resulted in a decrease in the pH of sausages and this decrease depended on the concentration of the plant additive. This is probably related to the fact that TP is characterized by high acidity (pH 4.48–5.02). Water activity in experimental raw fermented sausages ranged from 0.848 to 0.863. These results were lower compared to the results published in the previous experiment [25], which resulted in the inhibition of the growth of EC and EB, ensuring the microbiological stability of the meat product and thus not negatively affecting the development of LAB.

The influence of the addition of TP on the increase in the antioxidant activity of sausages was observed. This increase was also closely related to the concentration of the added plant additive. These observations were also confirmed by other researchers [36, 37]. The results obtained in this experiment were also similar to those published in our previous articles [25, 38]. However, contrary to previous studies, an opposite trend was observed, which showed that sausages analyzed after the cold storage process had higher antioxidant activity than those tested after the production process. This is a beneficial phenomenon because it allows the oxidation reactions to slow down and protects against their adverse effects on meat products.

In this context, it was shown that the addition of TP influenced the TBARS value in the experimental sausages, contributing to the delay of the fat oxidation and increasing the oxidative stability of meat products. This was most likely due to the fact that lycopene which is a strong antioxidant effectively neutralizes free oxygen radicals, including hydroxyl and peroxide radicals. These radicals are the main initiators of the fat oxidation reaction, which may reduce the rate of lipid oxidation in meat, and thus reduce the TBARS value. Moreover, polyphenols and vitamin C present in TP inhibit oxidation processes by interacting with metals, preventing their participation in reactions catalyzing fat oxidation. Lycopene, polyphenols, and vitamin C work synergistically, slowing down the development of peroxidation and preventing the formation of harmful oxidation products [24, 39]. Additionally, the obtained results ranged from 2.0 to 2.5 mg MDA kg^−1^ and did not exceed the level that results in the formation of unpleasant aroma and taste in meat and its products [40]. Similar observations were also reported by Candogan [41], Kim et al. [42], Kęska et al. [43], and Babaoğlu et al. [44]. The results obtained in our research indicated that the addition of by-products of the fruit and vegetable industry, including TP, inhibits the formation of primary and secondary lipid oxidation products in meat products. This effect can be attributed to lycopene and bioactive compounds (beta-carotene, polyphenols) contained in by-products, which contribute most to increasing the antioxidant activity of meat products. Additionally, the content of carotenoids (lycopene and *β*-carotene) in TP made it possible to obtain raw fermented sausages with reduced nitrite addition (up to 50 mg/kg) with a low TBARS value [45].

Protein oxidation, similar to lipid oxidation, is an important cause of meat quality deterioration. Our study found a significant effect (*p* ≤ 0.05) of the addition of TP on the content of carbonyl groups in the fermented sausages. They were characterized by a higher content of this parameter compared to the control sample. The results obtained differed from the observations of other authors [44, 46]. However, the effect of various plant additives on protein oxidation is not yet fully understood, unlike their effect on lipid oxidation. Many plant extracts such as blackcurrant, grapes, and pomegranate have an inhibitory effect on protein oxidation [47–49]. Studies by other authors have confirmed that the addition of rosemary and apple [50, 51] is ineffective in relation to the oxidation of proteins in various meat products. The different effectiveness of plant additives may result from different chemical structures of phenolic compounds [51].

Raw fermented sausages were characterized by a high content of some amino acids, mainly Asp (38.10–41.00 mg/g), Thr (21.40–22.40 mg/g), Glu (61.75–64.55 mg/g), Pro (29.75–31.95 mg/g), Leu (25.65–27.95 mg/g), Lys (22.55–25.10 mg/g), and Arg (20.80–22.60 mg/g), which confirms their nutritional value. Apart from a few cases, there was no significant effect (*p* ≤ 0.05) of the addition of TP on the amino acid content in the experimental sausages. In this study, the results obtained differed from those of other authors [52, 53]. In the current study, the most abundant amino acids were Asn and Glu. The possible reason for such high values of these amino acids in the analyzed products was the fact that the basic amino acids found in tomato seed protein include glutamic acid (19.44%–24.37%) and aspartic acid (8.82%–10.32%) which are a source of Glu and Asn [54].

In the experimental sausages, the LAB content ranged from 6.80 to 8.60 log CFU g^−1^. There was no significant effect of TP addition on the number of LAB in fermented sausages. These results were similar to those published in our previous studies [25, 38] and by other authors [44]. Microbiological analysis for mold and yeast content showed no significant differences between groups of tested meat products. Raw fermented sausages were characterized by low mold and yeast content (1.00–2.51 log CFU g^−1^). These results were lower compared to studies by other authors [55–57]. EC, EB, and STA were not detected in all samples (< 10 CFU g^−1^).

Statistical analysis showed that the addition of TP had a significant effect (*p* ≤ 0.05) on the color parameters of the final product. The *L*^∗^ value (lightness) of sausages decreased as a result of the increased concentration of TP. The results obtained are similar to those published in our previous paper [25] and by other authors [34, 35, 42, 58]. This phenomenon can be explained by the fact that TP is dark in color and adding it to meat products reduces the lightness of the color. It was observed that increasing the content of TP added to the tested experimental sausages also resulted in an increase in the *a*^∗^ and *b*^∗^ values, which is consistent with the results of Calvo et al. [58] and Ghafouri-Oskuei et al. [35]. The reason for the increase in the *a*^∗^ value in sausage samples with the addition of plant raw material can be attributed to lycopene, which is present in TP [59]. Although lycopene is a red pigment, its addition to meat may also change its color toward orange, which may be explained by the increase in the *b*^∗^ parameter in batches containing the addition of TP [34].

Statistical analysis showed a significant effect (*p* ≤ 0.05) of TP addition on the heme iron content in fermented sausages. It was found that with the increase in the addition of TP, the content of this parameter also increased. Statistically significant differences (*p* ≤ 0.05) were also observed in terms of storage time. All samples analyzed after production were characterized by a higher content of this parameter compared to sausages tested after 90 days of cold storage. The results obtained differed from those of other authors [60, 61]. The addition of TP rich in lycopene resulted in a lower release of iron bound in heme and thus a delay in the lipid oxidation, which was confirmed by the discussed earlier TBARS content results. This phenomenon could be due to the fact that lycopene and phenolic compounds present in TP can slow down the oxidation of hemoglobin. Additionally, they can increase the bioavailability of iron, especially that contained in heme, by breaking down protein complexes and improving its absorption by the body. It can therefore be assumed that their antioxidant properties and metal chelating ability may promote the release of iron from heme, thus affecting the overall quality of meat products [62, 63].

The TP used also significantly increased the hardness of the tested sausages. It was found that as the TP concentration increased, the hardness of meat products also increased. These observations were also confirmed by other authors [58, 64, 65]. This phenomenon is caused by the fact that TP contains large amounts of fiber, which modify the texture of meat products [64].

## 5. Conclusion

The research showed that the addition of TP increased the antioxidant activity of raw fermented sausages; therefore, it can be assumed that it enriched the products with lycopene and phenolic compounds that effectively inhibited oxidative changes in lipids. Fortification of sausages with TP also resulted in an increase of redness. Additionally, these sausages contained a higher amount of heme iron. However, the plant additive was not effective in inhibiting the protein oxidation in fermented sausage. Nevertheless, the obtained products were of high quality and TP had a positive impact on improving the characteristics of the meat product. The addition of TP increased the antioxidant activity of the tested sausages, which contributed to delaying fat oxidation, slowing down the processes of releasing iron bound in heme and increasing the oxidative stability of meat products and maintaining microbiological stability. Additionally, it improved the color of the final product, which may have a positive effect for consumer acceptance. It can therefore be concluded that TP in an amount as low as 2.5% can be used as a natural addition to the production of raw fermented sausages, thus enabling the reduction of nitrite consumption.

## Figures and Tables

**Table 1:** Proximate chemical composition (percentage) of dry fermented sausages.

**Parameter**	**Treatment**	**Storage time (days)**	
		**0**	**90**
Fat	SK	22.10 ± 0.56^aA^	21.30 ± 1.81^aA^
	STP 1.5%	21.01 ± 0.03^aA^	21.35 ± 0.22^aA^
	STP 2.5%	21.94 ± 0.07^aA^	21.87 ± 0.51^aA^

Protein	SK	43.72 ± 0.82^aA^	43.93 ± 2.05^aA^
	STP 1.5%	44.31 ± 0.02^aA^	41.89 ± 0.31^aA^
	STP 2.5%	44.05 ± 0.24^aA^	42.29 ± 0.03^aA^

Moisture	SK	30.69 ± 1.32^aA^	31.42 ± 0.72^aA^
	STP 1.5%	31.59 ± 0.22^aA^	31.90 ± 0.03^aA^
	STP 2.5%	30.29 ± 0.12^aA^	30.55 ± 0.65^aA^

Collagen	SK	4.34 ± 0.35^aB^	2.90 ± 0.32^aA^
	STP 1.5%	4.05 ± 0.02^aA^	3.44 ± 0.25^aA^
	STP 2.5%	4.29 ± 0.14^aA^	4.15 ± 0.43^aA^

Salt	SK	3.51 ± 0.07^bA^	3.94 ± 0.07^aB^
	STP 1.5%	3.45 ± 0.03^abA^	3.93 ± 0.14^aA^
	STP 2.5%	3.26 ± 0.00^aA^	3.88 ± 0.24^aA^

*Note:* SK—control sample; STP 1.5%—sample with 1.5% addition of TP; STP 2.5%—sample with 2.5% addition of TP. Means marked with the same lowercase letters a–b in the same column are not statistically different (*p* ≤ 0.05). Means marked with the same capital letter A–B in the same line (within the same variant of variable in different time) are not statistically different (*p* ≤ 0.05).

**Table 2:** pH and water activity of dry fermented sausages during processing.

**Parameter**	**Treatment**	**Storage time (days)**	
		**0**	**90**
pH	SK	4.51 ± 0.01^cA^	4.57 ± 0.01^bA^
	STP 1.5%	4.32 ± 0.01^bA^	4.38 ± 0.02^aB^
	STP 2.5%	4.27 ± 0.01^aA^	4.34 ± 0.01^aB^

Water activity	SK	0.848 ± 0.005^aA^	0.863 ± 0.002^bA^
	STP 1.5%	0.856 ± 0.003^abA^	0.862 ± 0.001^abA^
	STP 2.5%	0.860 ± 0.002^bA^	0.859 ± 0.001^aA^

*Note:* SK—control sample; STP 1.5%—sample with 1.5% addition of TP; STP 2.5%—sample with 2.5% addition of TP. Means marked with the same lowercase letters a–c in the same column are not statistically different from each other (*p* ≤ 0.05). Means marked with the same capital letter A–B in the same line (within the same variant of variable in different time) are not statistically different from each other (*p* ≤ 0.05).

**Table 3:** Antioxidant activity and oxidative stability of dry fermented sausages.

**Parameter**	**Treatment**	**Storage time (days)**	
		**0**	**90**
ABTS (mg Trolox eqv. g^−1^)	SK	0.049 ± 0.003^aA^	0.089 ± 0.006^aB^
	STP 1.5%	0.069 ± 0.003^bA^	0.117 ± 0.003^bB^
	STP 2.5%	0.073 ± 0.003^bA^	0.125 ± 0.002^bB^

DPPH (mg Trolox eqv. g^−1^)	SK	0.046 ± 0.003^aA^	0.068 ± 0.006^aB^
	STP 1.5%	0.080 ± 0.004^bA^	0.086 ± 0.002^bA^
	STP 2.5%	0.115 ± 0.017^cA^	0.120 ± 0.003^cA^

TBARS (mg MDA kg^−1^)	SK	2.44 ± 0.08^bB^	1.60 ± 0.20^bA^
	STP 1.5%	2.16 ± 0.04^aB^	1.27 ± 0.13^bA^
	STP 2.5%	2.06 ± 0.04^aB^	0.89 ± 0.09^aA^

Carbonyl content (nmole carbonyl mg protein^−1^)	SK	0.637 ± 0.015^aA^	0.451 ± 0.201^aA^
	STP 1.5%	0.681 ± 0.147^abA^	0.709 ± 0.075^aA^
	STP 2.5%	0.913 ± 0.067^bA^	1.378 ± 0.041^bB^

*Note:* SK—control sample; STP 1.5%—sample with 1.5% addition of TP; STP 2.5%—sample with 2.5% addition of TP. Means marked with the same lowercase letters a–c in the same column are not statistically different from each other (*p* ≤ 0.05). Means marked with the same capital letter A–B in the same line (within the same variant of variable in different time) are not statistically different from each other (*p* ≤ 0.05).

**Table 4:** Amino acid content in dry fermented sausages during processing (mg g^−1^).

**Parameter**	**Treatment**	**Storage time (days)**	
		**0**	**90**
Asp	SK	41.00 ± 0.56^aA^	40.00 ± 1.41^aA^
	STP 1.5%	39.65 ± 1.48^aA^	38.10 ± 0.14^aA^
	STP 2.5%	39.95 ± 0.63^aA^	38.40 ± 0.28^aA^

Thr	SK	22.40 ± 0.70^aA^	22.35 ± 1.06^aA^
	STP 1.5%	21.75 ± 0.77^aA^	21.55 ± 0.21^aA^
	STP 2.5%	21.40 ± 0.28^aA^	21.50 ± 0.14^aA^

Ser	SK	14.35 ± 0.35^aA^	14.15 ± 0.91^aA^
	STP 1.5%	13.95 ± 0.21^aA^	13.45 ± 0.07^aA^
	STP 2.5%	14.00 ± 0.14^aA^	13.80 ± 0.00^aA^

Glu	SK	64.55 ± 1.76^aA^	62.90 ± 3.81^aA^
	STP 1.5%	62.40 ± 1.83^aA^	61.75 ± 0.63^aA^
	STP 2.5%	62.80 ± 1.41^aA^	62.85 ± 0.63^aA^

Pro	SK	31.90 ± 1.41^aA^	31.95 ± 2.61^aA^
	STP 1.5%	31.80 ± 0.84^aB^	29.75 ± 1.06^aA^
	STP 2.5%	30.45 ± 1.20^aA^	29.80 ± 0.28^aA^

Gly	SK	16.45 ± 0.63^aA^	15.55 ± 0.77^aA^
	STP 1.5%	15.35 ± 0.21^aA^	14.90 ± 0.28^aA^
	STP 2.5%	15.90 ± 0.00^aA^	15.20 ± 0.14^aA^

Ala	SK	19.55 ± 0.77^aB^	18.80 ± 0.84^aA^
	STP 1.5%	18.60 ± 0.42^aA^	18.10 ± 0.14^aA^
	STP 2.5%	18.75 ± 0.07^aA^	18.30 ± 0.00^aA^

Val	SK	17.90 ± 0.56^aA^	17.35 ± 0.63^aA^
	STP 1.5%	17.35 ± 0.77^aA^	16.75 ± 0.35^aA^
	STP 2.5%	17.20 ± 0.28^aA^	16.70 ± 0.14^aA^

Ile	SK	16.85 ± 0.35^aA^	16.70 ± 0.70^aA^
	STP 1.5%	16.45 ± 0.63^aA^	15.95 ± 0.63^aA^
	STP 2.5%	16.50 ± 0.14^aA^	15.85 ± 0.07^aA^

Leu	SK	27.95 ± 0.91^aB^	26.70 ± 0.84^aA^
	STP 1.5%	26.30 ± 0.99^aA^	25.65 ± 0.07^aA^
	STP 2.5%	26.50 ± 0.28^aA^	26.15 ± 0.07^aA^

Tyr	SK	14.55 ± 0.63^aA^	14.50 ± 0.70^aA^
	STP 1.5%	14.10 ± 0.56^aA^	13.80 ± 0.14^aA^
	STP 2.5%	13.95 ± 0.07^aA^	14.15 ± 0.07^aA^

Phe	SK	14.65 ± 0.35^aA^	14.50 ± 0.70^aA^
	STP 1.5%	14.35 ± 0.49^aA^	13.95 ± 0.21^aA^
	STP 2.5%	14.25 ± 0.21^aA^	14.00 ± 0.00^aA^

His	SK	14.80 ± 0.56^aA^	14.55 ± 0.35^aA^
	STP 1.5%	14.10 ± 0.42^aA^	13.80 ± 0.14^aA^
	STP 2.5%	14.40 ± 0.00^aA^	14.20 ± 0.14^aA^

Lys	SK	25.10 ± 0.99^aA^	23.70 ± 0.56^aA^
	STP 1.5%	22.85 ± 0.49^aA^	22.55 ± 0.07^aA^
	STP 2.5%	24.10 ± 0.00^aA^	23.25 ± 0.35^aA^

Arg	SK	22.60 ± 0.99^aA^	22.00 ± 0.70^aA^
	STP 1.5%	22.15 ± 0.91^aA^	20.80 ± 0.28^aA^
	STP 2.5%	21.80 ± 0.28^aB^	21.10 ± 0.28^aA^

*Note:* SK—control sample; STP 1.5%—sample with 1.5% addition of TP; STP 2.5%—sample with 2.5% addition of TP. Means marked with the same lowercase letters a–c in the same column are not statistically different from each other (*p* ≤ 0.05). Means marked with the same capital letter A–B in the same line (within the same variant of variable in different time) are not statistically different from each other (*p* ≤ 0.05).

Abbreviations: Ala, alanine; Arg, arginine; Asp, asparagine; Glu, glutamine; Gly, glycine; His, histidine; Ile, isoleucine; Leu, leucine; Lys, lysine; Phe, phenylalanine; Pro, proline; Ser, serine; Thr, threonine; Tyr, tyrosine; Val, valine.

**Table 5:** Results of microbiological analyses of dry fermented sausages.

**Parameter**	**Treatment**	**Storage time (days)**	
		**0**	**90**
LAB (log CFU g^−1^)	SK	8.26 ± 0.21^aA^	7.73 ± 0.52^aA^
	STP 1.5%	8.55 ± 0.42^aB^	7.47 ± 0.10^aA^
	STP 2.5%	8.60 ± 0.06^aB^	6.80 ± 0.42^aA^

YM (log CFU g^−1^)	SK	1.65 ± 0.58^aA^	1.17 ± 0.29^aA^
	STP 1.5%	2.19 ± 0.13^aA^	1.53 ± 0.49^aA^
	STP 2.5%	2.51 ± 1.62^aA^	1.00 ± 0.00^aA^

EC (CFU g^−1^)	SK	< 10	< 10
	STP 1.5%	< 10	< 10
	STP 2.5%	< 10	< 10

EB (CFU g^−1^)	SK	< 10	< 10
	STP 1.5%	< 10	< 10
	STP 2.5%	< 10	< 10

STA (CFU g^−1^)	SK	< 10	< 10
	STP 1.5%	< 10	< 10
	STP 2.5%	< 10	< 10

*Note:* SK—control sample; STP 1.5%—sample with 1.5% addition of TP; STP 2.5%—sample with 2.5% addition of TP. Means with the same letter a–b do not differ significantly (*p* > 0.05) within the variable in the same day (column); means with the same cover letter A–B do not differ significantly (*p* > 0.05) within the same variant of variable in different day (row).

Abbreviations: EB, Enterobacteriaceae; EC, *E. coli*; LAB, lactic acid bacteria; STA, *Staphylococcus aureus*; YM, yeast and molds.

**Table 6:** CIE *L*^∗^, *a*^∗^, *b*^∗^ color parameters, heme iron content, and hardness of dry fermented sausages.

**Parameter**	**Treatment**	**Storage time (days)**	
		**0**	**90**
*L* ^∗^	SK	49.25 ± 3.29^aB^	47.95 ± 2.39^bA^
	STP 1.5%	48.99 ± 2.00^aB^	46.13 ± 1.15^abA^
	STP 2.5%	47.61 ± 1.71^aB^	44.96 ± 1.62^aA^

*a* ^∗^	SK	11.15 ± 0.58^aA^	11.18 ± 0.78^aA^
	STP 1.5%	20.85 ± 2.47^bA^	20.63 ± 1.13^bA^
	STP 2.5%	22.35 ± 1.72^bA^	22.86 ± 1.39^cA^

*b* ^∗^	SK	8.66 ± 0.56^aA^	9.11 ± 0.88^aA^
	STP 1.5%	21.42 ± 3.20^bA^	19.99 ± 2.56^bA^
	STP 2.5%	23.75 ± 3.12^bA^	21.82 ± 4.31^bA^

Δ*E*	SK		
	STP 1.5%	16.43 ± 3.74^aA^	14.80 ± 2.97^aA^
	STP 2.5%	18.92 ± 3.31^aA^	17.75 ± 2.19^aA^

Heme iron content (mg kg^–1^)	SK	15.99 ± 2.34^aA^	11.75 ± 0.12^aA^
	STP 1.5%	19.11 ± 1.13^abB^	12.63 ± 0.48^bA^
	STP 2.5%	24.58 ± 3.60^bB^	13.26 ± 0.14^bA^

Hardness (N)	SK	15.50 ± 6.23^aA^	22.40 ± 3.35^aB^
	STP 1.5%	21.78 ± 3.99^aA^	24.57 ± 4.07^aA^
	STP 2.5%	32.68 ± 8.81^bA^	29.41 ± 1.44^bA^

*Note:* SK—control sample; STP 1.5%—sample with 1.5% addition of TP; STP 2.5%—sample with 2.5% addition of TP. Means marked with the same lowercase letters a–b in the same column are not statistically different from each other (*p* ≤ 0.05). Means marked with the same capital letter A–B in the same line (within the same variant of variable in different time) are not statistically different from each other (*p* ≤ 0.05).

## Data Availability

The data that support the findings of this study are available from the corresponding author upon reasonable request.
